# Phenotype Harmonization in the GLIDE2 Oral Health Genomics Consortium

**DOI:** 10.1177/00220345221109775

**Published:** 2022-08-24

**Authors:** K. Divaris, S. Haworth, J.R. Shaffer, V. Anttonen, J.D. Beck, Y. Furuichi, B. Holtfreter, D. Jönsson, T. Kocher, S.M. Levy, P.K.E. Magnusson, D.W. McNeil, K. Michaëlsson, K.E. North, U. Palotie, P.N. Papapanou, P.J. Pussinen, D. Porteous, K. Reis, A. Salminen, A.S. Schaefer, T. Sudo, Y.Q. Sun, A.L. Suominen, T. Tamahara, S.M. Weinberg, P. Lundberg, M.L. Marazita, I. Johansson

**Affiliations:** 1 Division of Pediatric and Public Health, Adams School of Dentistry, University of North Carolina at Chapel Hill, Chapel Hill, NC, USA; 2 Department of Epidemiology, Gillings School of Global Public Health, University of North Carolina at Chapel Hill, Chapel Hill, NC, USA; 3 Medical Research Council Integrative Epidemiology United, Department of Population Health Sciences, Bristol Medical School, University of Bristol, Bristol, UK; 4 Bristol Dental School, University of Bristol, Bristol, UK; 5 Department of Human Genetics, Graduate School of Public Health, University of Pittsburgh, Pittsburgh, PA, USA; 6 Center for Craniofacial and Dental Genetics, Department of Oral and Craniofacial Sciences, School of Dental Medicine, University of Pittsburgh, Pittsburgh, PA, USA; 7 Research Unit of Oral Health Sciences, Faculty of Medicine, University of Oulu, Oulu, Finland; 8 Medical Research Center, Oulu University Hospital and University of Oulu, Oulu, Finland; 9 Division of Comprehensive Oral Health–Periodontology, Adams School of Dentistry, University of North Carolina at Chapel Hill, Chapel Hill, NC, USA; 10 Division of Endodontology and Periodontology, Department of Oral Rehabilitation, Graduate School of Dentistry, Health Sciences University of Hokkaido, Hokkaido, Japan; 11 Department of Restorative Dentistry, Periodontology, Endodontology, and Preventive and Pediatric Dentistry, University Medicine Greifswald, Greifswald, Germany; 12 Public Dental Service of Skåne, Lund, Sweden; 13 Hypertension and Cardiovascular Disease, Department of Clinical Sciences in Malmö, Lund University, Malmö, Sweden; 14 Faculty of Odontology, Malmö University, Malmö, Sweden; 15 Department of Preventive and Community Dentistry, College of Dentistry, University of Iowa, Iowa City, IA, USA; 16 Department of Medical Epidemiology and Biostatistics, Karolinska Institutet, Stockholm, Sweden; 17 Center for Oral Health Research in Appalachia, Appalachia, NY, USA; 18 Department of Psychology, West Virginia University, Morgantown, WV, USA; 19 Department of Dental Public Health & Professional Practice, West Virginia University, Morgantown, WV, USA; 20 Department of Surgical Sciences, Unit of Medical Epidemiology, Uppsala University, Uppsala, Sweden; 21 Carolina Population Center, University of North Carolina at Chapel Hill, Chapel Hill, NC, USA; 22 Oral and Maxillofacial Diseases, University of Helsinki and Helsinki University Hospital, Helsinki, Finland; 23 Division of Periodontics, Section of Oral, Diagnostic and Rehabilitation Sciences, Columbia University, College of Dental Medicine, New York, NY, USA; 24 Institute of Dentistry, School on Medicine, University of Eastern Finland, Kuopio, Finland; 25 Centre for Genomic and Experimental Medicine, Institute of Genetics and Cancer, University of Edinburgh, Edinburgh, UK; 26 Institute of Genomics, University of Tartu, Tartu, Estonia; 27 Department of Periodontology, Oral Medicine and Oral Surgery, Institute for Dental and Craniofacial Sciences, Charité–Universitätsmedizin Berlin, Berlin, Germany; 28 Institute of Education, Tokyo Medical and Dental University, Tokyo, Japan; 29 Center for Oral Health Services and Research Mid-Norway (TkMidt), Trondheim, Norway; 30 Department of Clinical and Molecular Medicine, NTNU, Norwegian University of Science and Technology, Trondheim, Norway; 31 Institute of Dentistry, School on Medicine, University of Eastern Finland, Kuopio, Finland; 32 Department of Oral and Maxillofacial Diseases, Kuopio University Hospital, Kuopio, Finland; 33 Public Health Evaluation and Projection Unit, Finnish Institute for Health and Welfare (THL), Helsinki, Finland; 34 Department of Community Medical Supports, Tohoku Medical Megabank Organization, Tohoku University, Sendai, Japan; 35 Department of Odontology, Section of Molecular Periodontology, Umeå University, Umeå, Sweden; 36 Department of Odontology, Section of Cariology, Umeå University, Umeå, Sweden

**Keywords:** Genetics, dental caries, data sciences, epidemiology, dentition, permanent, genome-wide association study

## Abstract

Genetic risk factors play important roles in the etiology of oral, dental, and craniofacial diseases. Identifying the relevant risk loci and understanding their molecular biology could highlight new prevention and management avenues. Our current understanding of oral health genomics suggests that dental caries and periodontitis are polygenic diseases, and very large sample sizes and informative phenotypic measures are required to discover signals and adequately map associations across the human genome. In this article, we introduce the second wave of the Gene-Lifestyle Interactions and Dental Endpoints consortium (GLIDE2) and discuss relevant data analytics challenges, opportunities, and applications. In this phase, the consortium comprises a diverse, multiethnic sample of over 700,000 participants from 21 studies contributing clinical data on dental caries experience and periodontitis. We outline the methodological challenges of combining data from heterogeneous populations, as well as the data reduction problem in resolving detailed clinical examination records into tractable phenotypes, and describe a strategy that addresses this. Specifically, we propose a 3-tiered phenotyping approach aimed at leveraging both the large sample size in the consortium and the detailed clinical information available in some studies, wherein binary, severity-encompassing, and “precision,” data-driven clinical traits are employed. As an illustration of the use of data-driven traits across multiple cohorts, we present an application of dental caries experience data harmonization in 8 participating studies (*N* = 55,143) using previously developed permanent dentition tooth surface–level dental caries pattern traits. We demonstrate that these clinical patterns are transferable across multiple cohorts, have similar relative contributions within each study, and thus are prime targets for genetic interrogation in the expanded and diverse multiethnic sample of GLIDE2. We anticipate that results from GLIDE2 will decisively advance the knowledge base of mechanisms at play in oral, dental, and craniofacial health and disease and further catalyze international collaboration and data and resource sharing in genomics research.

## Introduction

Oral diseases, mainly dental caries and periodontitis, affect approximately 3.5 billion people and are a major global burden of disease (Watt et al. 2020; Wen et al. 2021). Behavioral risk factors and social determinants of health are arguably the strongest influences on the development of common forms of oral disease (Peres et al. 2019). While upstream action and policy interventions are necessary to address these persistent diseases and associated health inequities, there is also a need to advance our understanding of the fundamental disease biology, which may help identify prime opportunities for intervention. To make headway in better diagnosing, predicting, and managing dental caries and periodontitis, we need to comprehensively characterize their genomic basis. To achieve this, the oral, dental, and craniofacial research community needs to leverage big data for discovery and translational applications. International collaboration and a focus on increasing diversity and inclusion of underrepresented populations (Popejoy and Fullerton 2016; Agler and Divaris 2020) are essential to make decisive advances in the genomics evidence base for oral and dental conditions.

The past decade has seen considerable activity in genomic studies of dental caries and periodontitis (Divaris 2019), and several recent reviews provide comprehensive summaries of the genomics evidence base to date (Nibali et al. 2019; Morelli et al. 2020). Despite these efforts, decisive advances in genomic discovery with practical implications have yet to be made in the oral health domain. Discovered genetic variants to date for dental caries explain less than 2% of the observed variance versus an estimated ~50% possibly explainable by genomics, and there are only a handful of consensus replicable loci for common oral diseases compared to hundreds for other common, complex diseases like type 2 diabetes (Kim et al. 2021). Moreover, the dental genomics literature mainly comprises reports from individual cohorts and participants of European ancestry. The Gene-Lifestyle Interactions in Dental Endpoints (GLIDE) consortium was the first global effort aimed at advancing the field of dental genomics via the formation of a broad international collaboration network (Shungin et al. 2015a). The first wave of GLIDE involved approximately half a million adult participants from 12 cohorts, 8 countries, and 3 continents and led to the discovery of 47 novel loci for dental caries (Shungin et al. 2019).

Successful examples of concerted international collaboration, data, and resource sharing in other genomics research areas include the Global Lipids Genetics Consortium (GLGC; Graham et al. 2021), Population Architecture using Genomics and Epidemiology (PAGE) Study (Shungin et al. 2015b), and Global Biobank (Zhou et al. 2021), among others. These consortia benefit from very large sample sizes numbering in the millions of participants. Naturally, the inclusion of very large numbers of study participants across many different underlying cohorts comes with unavoidable limitations, including logistical issues and scientific challenges (Stingone et al. 2017). The key scientific challenges usually involve harmonization of traits and analyses across studies with differences in population and sample characteristics, phenotype measurement or definition, and other methodological variations across contributing studies (Bennett et al. 2011).

Dental caries and periodontitis have unique properties that require additional careful consideration. Despite a vast diversity in clinical presentations, both diseases are defined at the individual level (International Classification of Diseases codes K02.xx and K05.xx) and can be initially described using binary “case status” definitions. This is a logical first step in phenotype selection and one that maximizes sample size across participating studies. However, there is considerable and arguably biologically informative variability within each dental caries or periodontitis case that is not captured by dichotomous classifications. Therefore, more refined, clinically, and biologically informed classifications are considered next, creating an unavoidable trade-off between clinical precision, interpretability, and power for genetic discovery (Agler, Shungin, et al. 2019). For the purposes of a genome-wide association study (GWAS), a data reduction step is necessary to convert detailed clinical information to analyzable traits—this can be done either by convention (e.g., a decayed, missing, and filled surfaces index) or using data-driven approaches. The question then becomes whether the latter approach is suitable and translatable across diverse populations with different oral disease experience. An equally important source of heterogeneity is tooth loss, which is itself a possible endpoint of both dental caries and periodontitis, with variable contributions across the age spectrum (Haworth et al. 2018a) that needs to be thoughtfully accounted for in the measurement of oral disease experience. Consideration of multiple traits, weighing theoretical assumptions, and incorporating empirical sensitivity analyses are all part of consortium GWAS. Rigor in these big data analyses is key, with each proposed phenotype having its own strengths and limitations, serving a different purpose in the quest for genomics discovery. Binary “naive” case status definitions will allow the maximum inclusion of cohorts and participants, offering gains in power; severity encompassing traits, available in fewer cohorts and participants, will leverage the recorded cumulative disease experience in a quantitative manner to identify risk-conferring variants; and caries patterns, available for a subset of cohorts, will leverage biologically informed disease subtypes to identify genetic signals underlying them.

In this article, we introduce GLIDE2, the evolution and expansion of the oral/dental genomics GLIDE consortium. First, we outline our strategy and rationale for big data harmonization in the study of dental caries following a 3-tiered phenotyping approach. We discuss challenges, opportunities, methodological considerations, and trade-offs emanating from the variation in available clinical information in the diverse participating cohorts. Then, we present an application of clinical dental caries experience data harmonization in GLIDE2 using previously developed permanent dentition dental caries pattern traits that are replicable and transferable across multiple population-based cohorts.

## Methods

The GLIDE consortium is an international collaborative effort investigating oral health genomics. Previous efforts undertaken by GLIDE have been reported in 2 recent publications that included up to 487,823 adults from 12 contributing studies (Shungin et al. 2019) and 19,003 children from 9 contributing studies (Haworth et al. 2018b). One key limitation of these studies is that the initial GLIDE efforts relied heavily on self-reported and proxy data for caries and periodontitis. For example, only 26,792 participants out of a total 487,823 contributed clinical dental examination data for caries experience (Shungin et al. 2019). The consortium’s expansion increases the diversity of participating cohorts. GLIDE2 comprises 21 studies, contributing upward of 700,000 participants for different dental caries or periodontitis analyses. All participating cohorts received ethics approvals by their local authorities and all participants provided written informed consent. In this article, we focus our presentation on data harmonization processes and applications related to dental caries (Table 1).

**Table 1.** Overview of the 21 Cohorts Contributing to the 3-Tiered Phenotyping Approach for Dental Caries Experience Analysis in Gene-Lifestyle Interactions in Dental Endpoints 2.

			Caries Traits Available for GWAS		
Cohort	Region	*n*	Prevalence	Severity	Patterns
ARIC	United States	5,527	✓	✓^a^	
CCDG: COHRA1/Dental SCORE	United States	1,810	✓	✓	✓
CCDG: COHRA2/COHRA Smile	United States	1,185	✓	✓	✓
CCDG: OFC1/OFC2	Africa, Asia, Europe, North America, South America	4,967	✓	✓^a,b^	
EstBB	Estonia	~200,000	✓		
FinnGen	Finland	~390,000	✓	✓^a^	
Generation Scotland	Scotland	~18,000	✓		
Health 2000/2011	Finland	7,831	✓	✓^a^	
HUNT4	Norway	4,933	✓	✓	✓
IFS	United States	253	✓	✓	✓
MDC/MOS	Sweden	11,176	✓	✓	✓
NFBC1966	Finland	1,483	✓	✓	
Parogene	Finland	508	✓	✓^a^	
Periogene North	Sweden	995	✓	✓	✓
SHIP START	Germany	3,362	✓^c^	✓	
SHIP TREND	Germany	944	✓^c^	✓^c^	✓
SIMPLER	Sweden	19,052	✓	✓	✓
SOL	United States	11,816	✓	✓	✓
TWINGENE/STR	Sweden	16,849	✓	✓	✓
ToMMo	Japan	5,360	✓		
VIKING	Sweden	3,823	✓	✓	✓
Total		709,874	709,874	486,514	72,836

GWAS, genome-wide association study.

a Tooth-level (i.e., decayed, missing, and filled teeth data) available only.

b Based on assessment of intraoral photographs.

c Based on half-mouth clinical examinations.

**Figure 1.**
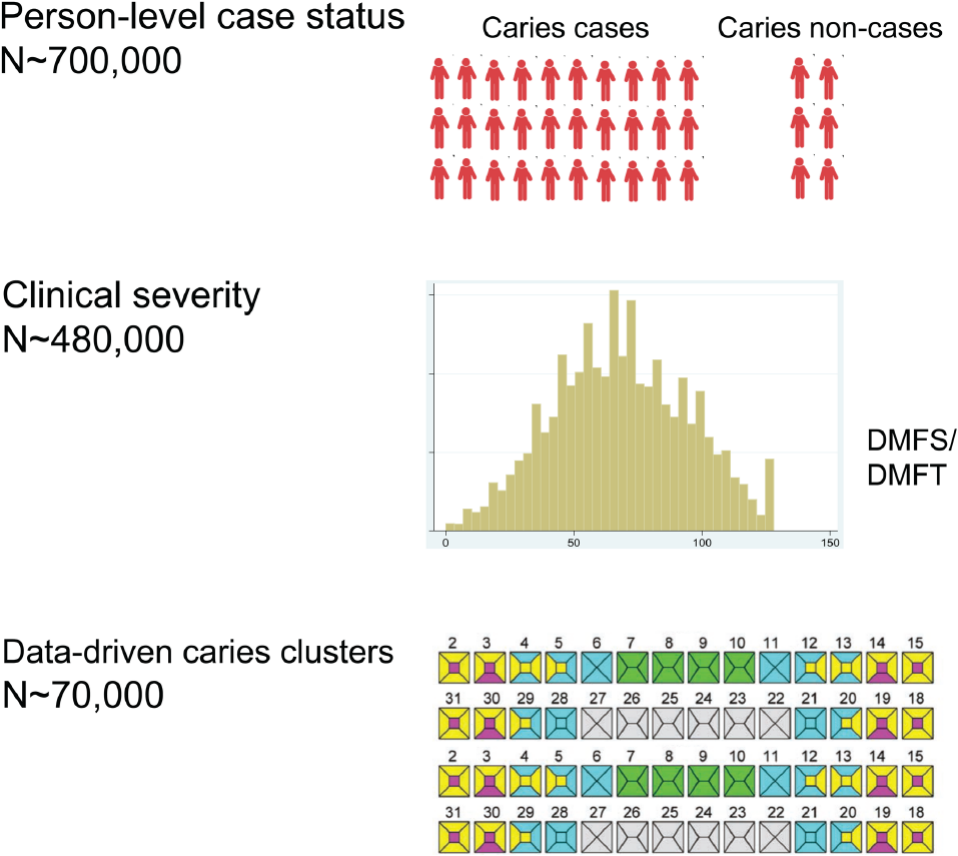
Illustration of the 3-level phenotyping definition strategy employed in Gene-Lifestyle Interactions in Dental Endpoints 2 (GLIDE2) for dental caries experience analysis. The maximum sample size is achieved for the relatively naive trait of binary caries case status (i.e., decayed, missing, filled teeth or surfaces [DMFT/DMFS] > 0). Second, we consider a quantitative measures of caries experience with demonstrated clinical relevance (i.e., DMFT/DMFS indices). Third, we employ data-driven tooth surface–level caries experience clusters that are available for a subset of participating studies.

Streamlining dental caries experience analyses on such a large scale, while a unique opportunity, can be daunting. First, variation exists in what has been measured and how in terms of caries experience (Appendix Supplemental Cohort summaries and Supplemental Methods). The overarching approach for phenotype harmonization in GLIDE2 is 3-tiered (Fig. 1). We begin by considering a broad definition of disease versus health (i.e., 1 or more decayed, missing, filled teeth or surfaces, DMFT/DMFS >0) to allow for the inclusion of the maximum number of participants from all contributing studies. Second, we consider a “consensus” quantitative measure of disease experience with demonstrated clinical relevance (i.e., DMFT/DMFS indices). Third, like previous genomics studies, we derive and plan to carry forward to GWAS data-driven “precision” dental traits. The latter are clinically and biologically informative patterns (i.e., clusters) of dental caries experience based on tooth surface–level data, according to the work of Shaffer, Feingold, Wang, Weeks, et al. (2013). These disease subtypes (e.g., pit-and-fissure caries experience versus smooth surface caries experience) likely reflect etiologic and biological differences (Shaffer, Feingold, et al. 2012; Shaffer, Wang, et al. 2012; Agler, Moss, et al. 2019) and are promising data-driven endpoints for genetic studies (Shaffer, Feingold, Wang, Lee, et al. 2013; Haworth et al. 2020), consistent with subtyping efforts undertaken for other common-complex diseases, including obesity (Field et al. 2013) and Parkinson’s disease (van Rooden et al. 2011). With this 3-tiered approach, we seek to leverage the unique features of GLIDE2: the case status analysis will maximize the sample size and statistical power, whereas the DMFS/DMFT quantitative analysis of caries experience will leverage information contained in disease severity, which is available for most cohorts. Finally, we will capitalize on all available tooth surface–level information on caries experience to carry out GWAS of permanent dentition caries clusters, which arguably contain more biological information than crude ones. To allow for the latter, it is imperative to understand whether these data-driven caries clusters generalize across cohorts.

In this study, we first examine demographic (i.e., age and sex) and clinical (caries experience and remaining natural teeth) characteristics of participants from 8 studies that contribute information to caries pattern explorations (Table 2). We anticipate that data from the remaining 13 studies will become available in the near future, although not all studies will contribute information on caries patterns—that is, we expect that ~72,000 participants will be included in this analysis, and thus our current sample is ~76% of the maximum target sample for this caries experience phenotype. These 8 studies are SIMPLER (Titova et al. 2021); STR (Zagai et al. 2019); MDC/MOS (Brunkwall et al. 2021); VIKING; COHRA1/Dental SCORE (Polk et al. 2008); COHRA2/COHRA Smile (Neiswanger et al. 2015); Periogene North, Iowa Fluoride Study (Wang et al. 2012); and OFC1/OFC2 (Leslie et al. 2016). The ascertainment of caries experience is harmonized at the moderate caries lesion threshold (International Caries Detection and Assessment System, ICDAS ≥3 or D_2_; Young et al. 2015), which is characterized by visible enable breakdown or signs of dentin demineralization. Teeth missing due to all causes are included in the calculation of the “M” component of the DMFS index, thereby creating a “tooth morbidity” DM_T_FS index in GLIDE2, consistent with previous genomics investigations (Shungin et al. 2019; Morelli et al. 2020). Our previous investigations among twins (Haworth et al. 2020) have showed that relative contributions from genetic and environmental factors are relatively stable over time in adulthood—justifying the combination of standardized estimates emanating from cohorts of different ages in the planned meta-analyses. Detailed information about the participating cohorts, parent studies and populations, methods, and phenotype and genotype data availability is presented in the appendix (Appendix Table 1).

**Table 2.** Demographic and Clinical Characteristics of Participants in 8 Cohorts Contributing to Dental Caries Clusters Harmonization.

Cohort	*N*	Demographics		Natural Teeth		Binary Caries Case Status	Quantitative Caries Experience, Mean (SD)		Tooth Surface–Level Caries Clusters (Shaffer, Feingold, Wang, Weeks, et al. 2013), Mean (SD)				
		Age, Mean (SD), y	Women, %	Edentulous, %	No. of Teeth, Mean (SD)	DM_T_FS/T >0, *n* (%)	DM_T_FT	DM_T_FS	Cluster 1	Cluster 2	Cluster 3	Cluster 4	Cluster 5
SIMPLER	19,052	73.6 (8.0)	33.7	1.4	23.2 (6.3)	17,416 (91.4)	14.8 (8.8)	55.0 (35.4)	0.65 (0.35)	0.14 (0.28)	0.58 (0.33)	0.39 (0.38)	0.38 (0.32)
TWINGENE/STR	16,849	48.7 (19.0)	58.2	0.3	26.0 (3.9)	15,893 (94.3)	12.3 (7.9)	35.7 (31.4)	0.56 (0.35)	0.06 (0.18)	0.40 (0.33)	0.21 (0.30)	0.22 (0.27)
MDC/MOS	11,176	67.9 (17.9)	63.4	0.9	23.6 (5.9)	10,874 (97.3)	17.8 (7.5)	62.9 (34.2)	0.74 (0.31)	0.17 (0.28)	0.65 (0.32)	0.45 (0.38)	0.46 (0.33)
VIKING	3,823	63.8 (8.0)	63.4	0.9	24.7 (4.9)	3,772 (98.7)	17.3 (7.8)	55.9 (31.7)	0.73 (0.31)	0.13 (0.23)	0.60 (0.31)	0.38 (0.34)	0.37 (0.30)
CCDG: COHRA1/Dental SCORE	1,810	43.8 (15.7)	64.6	5.1	23.1 (7.2)	1,763 (97.4)	13.7 (7.3)	44.1 (34.3)	0.73 (0.28)	0.10 (0.25)	0.46 (0.34)	0.29 (0.37)	0.26 (0.31)
CCDG: COHRA2/COHRA Smile	1,185	32.4 (6.2)	100	0.7	26.4 (3.7)	1,109 (93.6)	8.7 (6.4)	22.7 (23.7)	0.53 (0.33)	0.03 (0.12)	0.24 (0.46)	0.13 (0.25)	0.11 (0.20)
Periogene North	995	49.0 (13.1)	57.6	0	25.6 (3.8)	951 (95.6)	12.0 (7.5)	34.1 (29.8)	0.58 (0.32)	0.07 (0.18)	0.38 (0.31)	0.20 (0.30)	0.19 (0.26)
IFS	253	22.7 (1.8)	56.5	0	24.7 (2.9)	243 (96.0)	4.0 (3.3)	7.1 (8.9)	0.23 (0.24)	0.01 (0.04)	0.05 (0.09)	0.03 (0.09)	0.05 (0.12)

**Figure 2.**
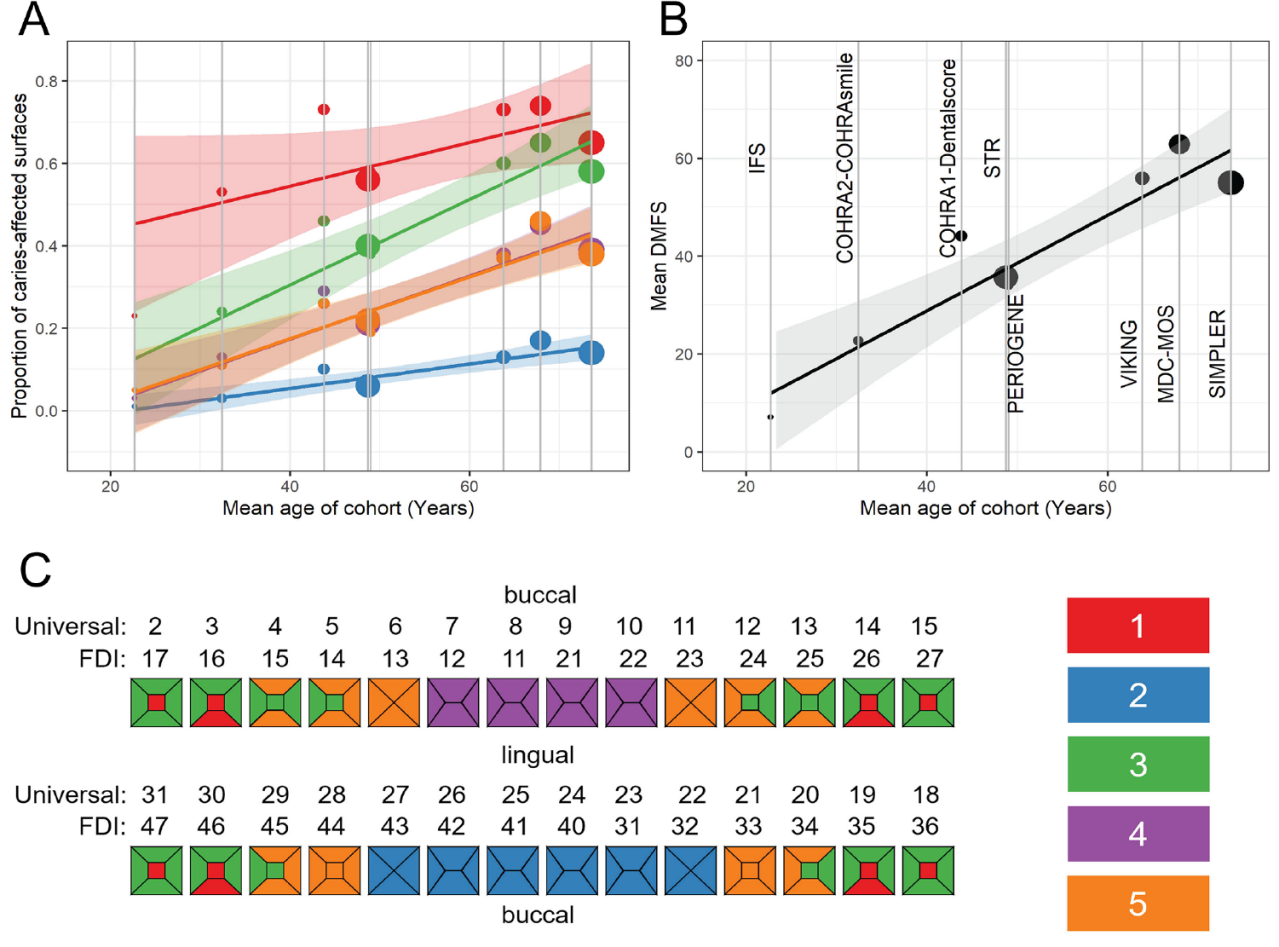
Caries experience (defined as the mean proportion of caries-affected surfaces within each cluster) differs among the 5 caries clusters in Gene-Lifestyle Interactions in Dental Endpoints 2 (GLIDE2) with similar patterns across all GLIDE2 cohorts. Caries experience in these caries clusters increases with age in the GLIDE2 cohorts (**A**), mirroring the overall increase in decayed, missing, and filled surfaces (DMFS) with age. (**B**) The size of markers is scaled to the number of participants in the participating studies. Regression lines and standard errors are estimated from inverse standard error-weighted linear meta-regression models. Cluster membership is illustrated on the odontogram (**C**), and colors in the legend refer to the cluster numbers given in Table 2.

Mean and standard deviation (SD) of caries experience are presented for each cluster, computed as the cluster-specific decayed, missing, and filled surfaces (DMFS) divided by the number of tooth surfaces in the cluster. The labeling of caries clusters corresponds to the nomenclature of Shaffer, Feingold, Wang, Weeks, et al. (2013) as follows: cluster 1, molar pits and fissures; cluster 2, lower anterior teeth; cluster 3, molar smooth surfaces, premolar pits, and proximal surfaces; cluster 4, maxillary incisors; and cluster 5, maxillary canines and premolar smooth surfaces. A visual representation of surfaces contributing to these clusters is presented in Figure 2, and the exact derivation is presented in Appendix Table 2. DM_T_FS, surface level tooth morbidity index; DM_T_FT, tooth level tooth morbidity index.

The caries experience clusters employed in this study were first introduced by Shaffer, Feingold, Wang, and Weeks (2013), who used hierarchical clustering of tooth surface–level information from all permanent teeth excluding third molars to identify 5 clusters of tooth surfaces with distinct patterns of caries experience. The existence of these clusters was verified in the National Health and Nutrition Examination Survey (NHANES, 1999–2000) data (Shaffer, Feingold, Wang, Weeks, et al. 2013) and in the Swedish GLIDE2 cohorts. In this article, we do not derive these clusters de novo but rather use the clusters definitions reported in Shaffer, Feingold, Wang, and Weeks (2013) to “score” each participating study, by adding surface-level caries experience data into 5 predefined groups of tooth surfaces (e.g., pits and fissures on molars) (Appendix Table 2). We represent these patterns of caries experience using color-coded odontograms (i.e., annotated representations of the permanent dentition and investigate between-cohort differences). Finally, we conduct power analyses, comparing GLIDE2 with the first wave of GLIDE with clinical data. Data management, analyses, and figure creation were done using SAS version 9.4 (SAS Institute).

## Results

Twenty-one studies (Table 1) contributed dental caries experience data in GLIDE2, a combined sample size of over 700,000 participants. As expected, the maximum sample size is available for binary case status analyses. Most studies (18/21) have quantitative caries experience information in the form of the DMFT or DMFS index. Eleven studies are expected to contribute tooth surface–specific data on caries experience, allowing for the application of the third level of data-driven caries clusters. Here, we present information for 8 of these cohorts that, as of February 2022, have contributed data from 55,143 adults (Table 2).

Demographic differences were evident in the analytic sample, both in terms of sample size and age. For example, the mean age was 74 y among 19,052 individuals in SIMPLER versus 23 y among 253 individuals in the IFS. COHRA2 is a female-only sample while the other studies contained both male and female participants. The prevalence of edentulism ranged from under 1% in the youngest samples (i.e., COHRA2 and IFS) to over 5% in COHRA1, and the average number of remaining natural teeth (excluding third molars) ranged between 23 and 26. Across the consortium, most participants had caries experience (DMFT/DMFS >0), but there was an appreciable number of participants who were caries free based on the study’s case definition, that is, 5.7% (*n* = 3,122 of 55,143) in the 8 studies included here. Differences were also evident in quantitative measures of caries experience, with high mean DMFS indices (above 55) in SIMPLER, MDC/MOS, and VIKING versus low mean DMFS (under 25) for COHRA2 and IFS.

We found that within-cluster caries experience paralleled the overall caries experience within each study, as well as participants’ mean age. The relative contribution (i.e., ordered rank) of each cluster was remarkably consistent across studies, with posterior teeth (2 clusters involving molars and premolars) contributing the highest and lower incisors exhibiting the lowest caries experience (Table 2). As expected, overall and within-cluster caries experience was lower among younger compared to older samples (Fig. 2). Nevertheless, tooth surfaces with the highest susceptibility (i.e., molar pits and fissures) were consistent across cohorts, regardless of background caries rate.

**Figure 3.**
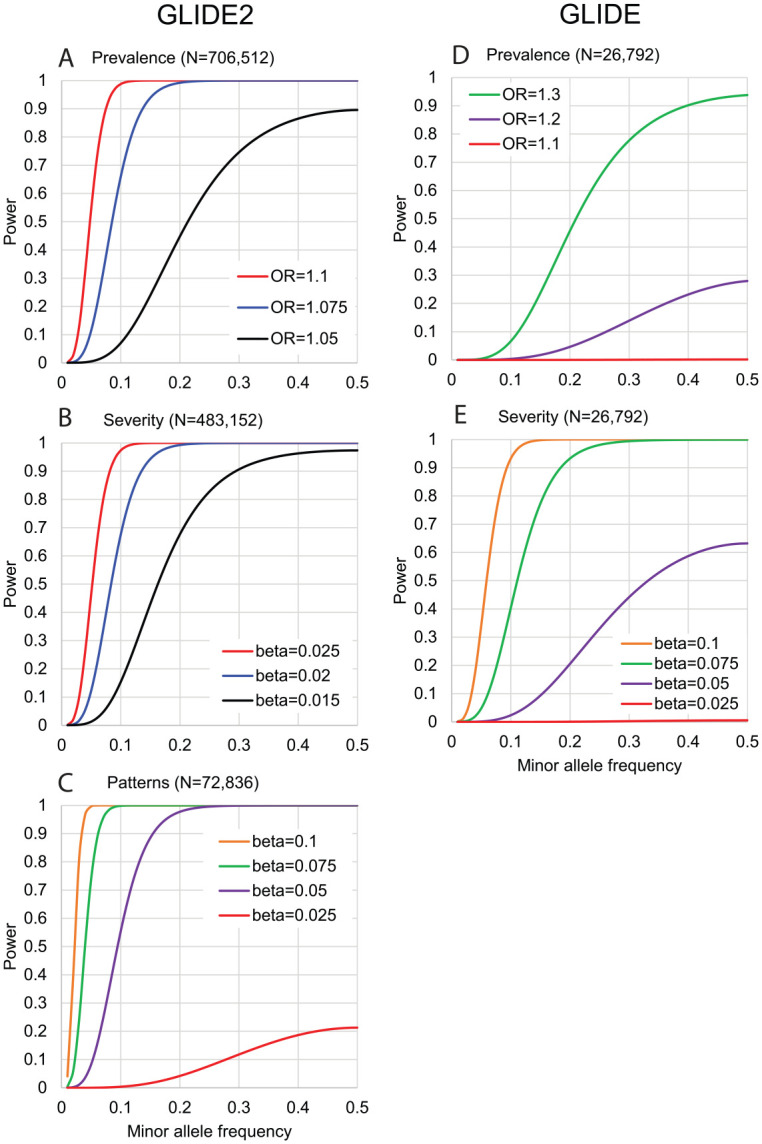
Power estimates in GLIDE2 versus GLIDE. Power (y-axis) to detect genetic association in (**A**–**C**) the Gene-Lifestyle Interactions in Dental Endpoints 2 (GLIDE2) consortium and (**D**–**E**) the original GLIDE sample with available clinical data, for a range of effect sizes (odds ratio [OR] for caries prevalence, β coefficient [i.e., per allele difference in units of trait standard deviation] for caries severity and patterns] across a spectrum of minor allele frequencies (x-axis).

Power estimates (Fig. 3) demonstrate that GLIDE2 has greater statistical power than GLIDE to detect caries-associated genetic variants with small effect sizes. For caries severity, we estimate GLIDE2 will have 80% power to detect individual variants each explaining 0.008% (i.e., less than one-hundredth of a percent) of variation in caries experience.

## Discussion

In this article, we introduced GLIDE2, the second study proposed by our international oral/dental genomics consortium, with improved clinical phenotypes, larger sample size, and greater diversity than previous studies. We discussed the key challenges of interrogating the genomics of dental, oral, and craniofacial diseases in an international consortium and considered options to harmonize phenotypic data. We outlined a 3-tiered phenotyping approach, including naive binary disease definitions to maximize sample size, quantitative caries experience indices, and data-driven, precision phenotypes encoding dental caries experience within distinct permanent dentition tooth surface clusters. We demonstrated that despite the unavoidable heterogeneity in population demographics and caries experience, these data-driven patterns are generalizable across the examined study populations and thus can be carried forward to GWAS meta-analyses in a larger group of GLIDE2 participating studies. We posit that this is justifiable even in the common scenario where clinical examination protocols and conditions differ. These unmodeled sources of variation contribute to unavoidable trait heterogeneity between studies and may reduce power to detect true signals. However, as long as clinical data are valid measures of the oral disease or endpoint under analysis, these differences are unlikely confounders of genetic associations (i.e., they will not generate spurious ones). We demonstrate that, using the approach described above, GLIDE2 will have unprecedented statistical power to discover genetic risk loci with modest effects on oral diseases, an important feature given their polygenic genetic architectures. Even if some of the identified variants may explain small proportions of disease variance, they can have profound impacts on disease biology and offer targets for prevention and therapy; for example, GWASs identified in *HMGCR* and *PCSK9* may explain little phenotypic variance (Lu et al., 2017) but are very important targets for cardiovascular disease prevention (Ference et al. 2016).

A key element of GLIDE2 is increased diversity and inclusion of underrepresented populations, with the representation of multiethnic populations and studies conducted in Africa, Asia, Europe, and North and South America. However, clinical examination data from traditionally underrepresented areas are still limited. The OFC1/OFC2 studies that include the most diverse representation are based on intraoral photographs and thus indirect assessments of dental health at the tooth level. Thus, there is still a need to encourage genomics studies of oral health and disease among populations and global regions that are currently underrepresented. Inclusion of multiethnic population samples should improve our ability to fine-map association signals and enable the development of transferrable polygenic risk scores (Graham et al. 2021), especially due to the enhanced ability to detect even small-in-magnitude signals for dental caries experience, periodontitis, and tooth loss. We will not employ a discovery-replication design, and all cohorts will contribute to the discovery of genetic signals—but we will use methods such as MAMBA (Meta-Analysis Model-based Assessment of replicability) (McGuire et al. 2021) that examine the distribution of genetic effects to identify variants that are potentially nonreplicable and those with high posterior probability for replication.

Despite the variation in dental disease experience inherent in an international consortium, the data presented in this article show it is feasible to harmonize traits and enable a well-powered GWAS. While this article has focused on dental caries experience, the challenges and possible solutions are similar for periodontitis. Obviously, the maximum sample size will be only available for relatively naive traits of dental caries and periodontitis (i.e., binary case definitions). Accounting for disease severity will likely offer advantages in statistical power for discovery while maintaining a sizable analytical sample. Leveraging caries clusters, as demonstrated in this article, is an important addition to available analytic endpoints, especially if genetic variant effects differ across clusters. These data-driven clusters were found to be consistent in terms of relative contribution across cohorts. In a recent study among a large sample of up to 41,678 Swedish twins, a similar but slightly different cluster solution was identified (Haworth et al. 2020). Despite some expected variation that would emerge if each cohort rederived their own data-driven cluster solution, we have found that the use of a “consensus” 5-level solution results in appreciable homogeneity, while these clusters have been shown to be clinically as well as biologically informative.

The inherent heterogeneity in population ancestry in GLIDE2 is likely to influence results. While this could initially be seen as a limitation, we posit that it is a relative strength and an opportunity that can be leveraged analytically. In a multiethnic meta-analysis, highest power will be obtained for signals that are homogeneous across ancestral populations, while signals that are heterogeneous would be harder to discover. On the other hand, multiethnic samples could allow for better fine-mapping of association signals in risk loci and help produce more informative and representative polygenic risk scores. The GWAS results can also form the substrate for a second tier of harmonization to further boost power by adjusting away differences in measurement between traits (Luningham et al. 2019), borrowing information across traits using multitrait analysis of GWAS summary statistics. In addition, we expect that GLIDE2 results will inform Mendelian randomization studies and other explorations of shared biology between oral and systemic health traits. All these advanced post-GWAS strategies will rely on the well-conducted, carefully phenotyped, adequately powered, and informative “basic” GLIDE2 GWAS. Geared toward transparency, reproducibility, and value creation for the community (Schwendicke et al. 2022), GLIDE2 summary results will be publicly shared, like the publicly deposited first GLIDE study results (https://data.bris.ac.uk/data/).

In conclusion, data-driven approaches are both suitable and necessary for the purposes of harmonization of oral health endpoints in large-scale, consortium-level applications such as GLIDE2. There are unavoidable trade-offs between detailed clinical measures and power for genetic discovery—to overcome those, we propose the utilization of multiple, complementary approaches for trait harmonization. We anticipate that results from GLIDE2 will advance the knowledge base of mechanisms at play in oral, dental, and craniofacial health and disease and further catalyze international collaboration and data and resource sharing in genomics research.

## Author Contributions

K. Divaris, S. Haworth, J.R. Shaffer, M.L. Marazita, I. Johansson, contributed to conception, design, and data acquisition, drafted and critically revised the manuscript; V. Anttonen, J.D. Beck, Y. Furuichi, B. Holtfreter, D. Jonsson, T. Kocher, S.M. Levy, P.K.E. Magnusson, D.W. McNeil, K. Michaelsson, K.E. North, U. Palotie, P.N. Papapanou, P.J. Pussinen, D. Porteus, K. Reis, A. Salminen, A.S. Schaefer, T. Sudo, Y.Q. Sun, A.L. Suominen, T. Tamahara, S.M. Weinberg, P. Lundberg, contributed to data acquisition, critically revised the manuscript. All authors gave final approval and agree to be accountable for all aspects of the work.

## Supplemental Material

sj-docx-1-jdr-10.1177_00220345221109775 – Supplemental material for Phenotype Harmonization in the GLIDE2 Oral Health Genomics Consortium Click here for additional data file. Supplemental material, sj-docx-1-jdr-10.1177_00220345221109775 for Phenotype Harmonization in the GLIDE2 Oral Health Genomics Consortium by K. Divaris, S. Haworth, J.R. Shaffer, V. Anttonen, J.D. Beck, Y. Furuichi, B. Holtfreter, D. Jönsson, T. Kocher, S.M. Levy, P.K.E. Magnusson, D.W. McNeil, K. Michaëlsson, K.E. North, U. Palotie, P.N. Papapanou, P.J. Pussinen, D. Porteous, K. Reis, A. Salminen, A.S. Schaefer, T. Sudo, Y.Q. Sun, A.L. Suominen, T. Tamahara, S.M. Weinberg, P. Lundberg, M.L. Marazita and I. Johansson in Journal of Dental Research
