# Supplementary material for: Phenotype Harmonization in the GLIDE2 Oral Health Genomics Consortium
Source: J Dent Res. 2022 Aug 24;101(11):1408–16. doi: 10.1177/00220345221109775 (PMC9516613; doi:10.1177/00220345221109775)
Supplement: sj-docx-1-jdr-10.1177_00220345221109775 – Supplemental material for Phenotype Harmonization in the GLIDE2 Oral Health Genomics Consortium [file sj-docx-1-jdr-10.1177_00220345221109775.docx]

**APPENDIX**

Kimon Divaris^1,2^, Simon Haworth^3,4^, John R. Shaffer^5,6^, Vuokko Anttonen^7,8^, James D. Beck^9^, Yasushi Furuichi^10^, Birte Holtfreter^11^, Daniel Jönsson^12-14^, Thomas Kocher^11^, Steven M. Levy^15^, Patrik K.E. Magnusson^16^, Daniel W. McNeil^17-19^, Karl Michaëlsson^20^, Kari E. North^2,21^, Ulla Palotie,^22^ Panos N. Papapanou^24^, Pirkko J. Pussinen^22,23^, David Porteous^25^, Kadri Reis^26^, Aino Salminen,^22^ Arne S. Schaefer^27^, Takeaki Sudo^28^, Yi-Qian Sun^29,30^, Anna Liisa Suominen^23,31,32^, Toru Tamahara^33^, Seth M. Weinberg^5,6^, Pernilla Lundberg^34^, Mary L. Marazita^5,6^, Ingegerd Johansson^35^*

^1^ Division of Pediatric and Public Health, Adams School of Dentistry, University of North Carolina at Chapel Hill, Chapel Hill, NC, USA

^2^ Department of Epidemiology, Gillings School of Global Public Health, University of North Carolina at Chapel Hill, Chapel Hill, NC, USA

^3^ Medical Research Council Integrative Epidemiology United, Department of Population Health Sciences, Bristol Medical School, University of Bristol, Bristol, UK

^4^ Bristol Dental School, University of Bristol, Bristol, UK

^5^ Department of Human Genetics, Graduate School of Public Health, University of Pittsburgh, Pittsburgh, PA, USA

^6^ Center for Craniofacial and Dental Genetics, Department of Oral and Craniofacial Sciences, School of Dental Medicine, University of Pittsburgh, Pittsburgh, PA, USA

^7^ Research Unit of Oral Health Sciences, Faculty of Medicine, University of Oulu, Oulu, Finland

^8^ Medical Research Center, Oulu University Hospital and University of Oulu, Oulu, Finland

^9^ Division of Comprehensive Oral Health - Periodontology, Adams School of Dentistry, University of North Carolina at Chapel Hill, Chapel Hill, NC, USA

^10^ Division of Endodontology and Periodontology, Department of Oral Rehabilitation, Graduate School of Dentistry, Health Sciences University of Hokkaido, Hokkaido, Japan

^11^ Department of Restorative Dentistry, Periodontology, Endodontology, and Preventive and Pediatric Dentistry, University Medicine Greifswald, Greifswald, Germany

^12^ Public Dental Service of Skåne, Lund, Sweden

^13^ Hypertension and Cardiovascular Disease, Department of Clinical Sciences in Malmö, Lund University, Malmö, Sweden

^14^ Faculty of Odontology, Malmö University, Malmö, Sweden

^15^ Department of Preventive and Community Dentistry, College of Dentistry, University of Iowa, Iowa City, Iowa, USA

^16^ Department of Medical Epidemiology and Biostatistics, Karolinska Institutet, Stockholm, Sweden

^17^ Center for Oral Health Research in Appalachia, Appalachia, New York, USA

^18^ Department of Psychology, West Virginia University, Morgantown, West Virginia, USA

^19^ Department of Dental Public Health & Professional Practice, West Virginia University, Morgantown, West Virginia, USA

^20^ Department of Surgical Sciences, Unit of Medical Epidemiology, Uppsala University, Uppsala, Sweden

^21^ Carolina Population Center, University of North Carolina at Chapel Hill, Chapel Hill, North Carolina, USA

^22^ Oral and Maxillofacial Diseases, University of Helsinki and Helsinki University Hospital, Helsinki, Finland

^23^ Institute of Dentistry, School on Medicine, University of Eastern Finland, Kuopio, Finland

^24^ Division of Periodontics, Section of Oral, Diagnostic and Rehabilitation Sciences, Columbia University, College of Dental Medicine, New York, NY, USA

^25^ Centre for Genomic and Experimental Medicine, Institute of Genetics and Cancer, University of Edinburgh, Edinburgh, UK

^26^ Institute of Genomics, University of Tartu, Tartu, Estonia

^27^ Department of Periodontology, Oral Medicine and Oral Surgery, Institute for Dental and Craniofacial Sciences, Charité - Universitätsmedizin Berlin

^28^ Institute of Education, Tokyo Medical and Dental University, Tokyo, Japan

^29^ Center for Oral Health Services and Research Mid-Norway (TkMidt), Trondheim, Norway

^30^ Department of Clinical and Molecular Medicine, NTNU, Norwegian University of Science and Technology, Trondheim, Norway

^31^ Institute of Dentistry, School on Medicine, University of Eastern Finland, Kuopio, Finland

^32^ Department of Oral and Maxillofacial Diseases, Kuopio University Hospital, Kuopio, Finland

^32^ Public Health Evaluation and Projection Unit, Finnish Institute for Health and Welfare (THL), Helsinki, Finland

^33^ Department of Community Medical Supports, Tohoku Medical Megabank Organization, Tohoku University, Sendai, Japan

^34^ Department of Odontology, Section of Molecular Periodontology, Umeå University, Umeå, Sweden

^35^ Department of Odontology, Section of Cariology, Umeå University, Umeå, Sweden

**Corresponding author**

Ingegerd Johansson, Department of Odontology, Section of Cariology, Umeå University, Umeå, Sweden. Email: [ingegerd.johansson@umu.se](mailto:ingegerd.johansson@umu.se)

**Supplemental Cohort summaries**

**SIMPLER: Swedish Infrastructure for Medical Population-Based Life-Course and Environmental Research.** The SIMPLER cohort includes all women born in 1914 to 1948 and men born in 1914 to 1952 living in two counties in middle Sweden. In total, the cohort has approximately 107,000 participants with up to six panels with questionnaire data, information on anthropometric and basic medical screening measures, a biobank and data on shot gun characterization of gut microbiota, plasma metabolomics and screening. Dental data were obtained from the Swedish quality register for caries and periodontitis (SKaPa). SIMPLER was supported by funding from the Swedish Research Council grants no 2017-06100 and 2017-00644. The dental part was supported by the Swedish Research Council grant no 2020-00930.

**STR: The Swedish Twin Registry** is a research infrastructure located at Karolinska Institutet, Stockholm. Sweden. The STR has information on twins born in Sweden 1886 or later. All twins born in Sweden are systematically invited when they turn 9-years of age. Questionnaire information and samples (blood or saliva) for DNA extraction are collected. The register has about 90,000 twin pairs with known zygosity. Dental data were obtained from the Swedish quality register for caries and periodontitis (SKaPa). The Swedish Twin Registry was supported by the Swedish Research Council grants no. 2017-00641. The dental part was supported by the Swedish Research Council grants no 2015-02597 and 2020-00930.

**MDC/MOS** recruited participants in the southern part of Sweden and in the **Malmö Diet-Cancer (MDC) study,** 17,000 women (born 1923-1950) and 11,000 men (born 1923-1945). Information on lifestyle, socio-economic factors, medications and past illnesses was obtained through questionnaires and a blood-based biobank was created. The **Malmö Offspring Study (MOS)** recruited 5,259 participants between 2013 and 2021. The study intends to map family patterns behind the large endemic diseases based on gene-environment interactions (Manjer et al., 2001). MOS has an optional dental study (MODS) with clinical assessment of caries and periodontitis measures but to obtain per tooth surface information dental data were also obtained from the Swedish quality register for caries and periodontitis (SKaPa). MDC and MOS were supported by the Swedish Research Council grant no. 521-2013-2756 and the Heart and Lung Foundation (Grant 20150427) and funds from the local Region Skåne County Council (ALF). MODS was supported by Oral Health Related Research by Region Skåne. The dental part I GLIDE was supported by the Swedish Research Council grant no 2020-00930 for both cohorts and also grant no 2015-02597 for MDC.

**The VIKING** cohort is nested in the Northern Sweden Health and Disease Study and specifically in the Västerbotten Intervention program arm. VIKING was initiated to study genetic risk assessment for high blood lipid levels and sudden death due to cardiovascular diseases with a genealogic approach by linking to the Swedish Demographic database at Umeå University with information on 1,5 million people and their families starting in the 18^th^ century. Dental data were obtained from the Swedish quality register for caries and periodontitis (SKaPa). The VIKING cohort was supported by the Swedish Research Council grant no 2020-00930.

**Periogene North.** Periogene North is a case-control study with questionnaire information and blood in a biobank for 1,059 participants aged 20 to 88 years. Periodontal status was assessed in Periogene but to obtain per tooth surface information, dental data were also obtained from the Swedish quality register for caries and periodontitis (SKaPa). Periogene North was supported by the Swedish Research Council grant no. 2020-00930.

**CCDG: COHRA1/ Dental SCORE and COHRA2/COHRA Smile**

The COHRA1/ Dental Strategies Concentrating on Risk Evaluation (SCORE) (Polk et al. 2008) and COHRA2/COHRA Smile (Neiswanger et al. 2015) cohorts come from the University of Pittsburgh Center for Craniofacial and Dental Genetics (CCDG)´s, Center for Oral Health Research in Appalachia (COHRA). The COHRA collaboration between the University of Pittsburgh and West Virginia University was designed to address oral health disparities in the Appalachian region in the United States, which has the largest burden of oral health problems per capita in the United States. Participants of all ages are included in the cohorts, but for the current study only adults 18 years or older were included. The participants were recruited from rural Appalachia in West Virginia and Southwestern Pennsylvania by using a household-based recruitment protocol and using the same data collection protocols for every site. For COHRA1/ Dental SCORE, recruitment, and data collection, including the dental data, started in 2003 and ended in 2009. Training and calibration for the periodontal and dental exam was done at the University of Pittsburgh for all sites at the beginning of the data collection and periodically during the collection of the data. The dental examination was performed by trained and calibrated dental examiners (either a dentist or dental hygienist plus an assistant). The assessments were calibrated at the start of the study and during data collection. COHRA2/ COHRA Smile recruitment and follow-up started in 2011 and are ongoing. Recruited women were from West Virginia and southwest Pennsylvania who were in their first and second trimesters of pregnancy. They entered longitudinal follow-up of the women and their offspring until the children reached at least age six. Data were collected through multiple in-person interviews, periodic telephone questionnaires, samples for DNA extraction, and dental assessment. IRB approvals for the COHRA cohorts were obtained at both the University of Pittsburgh (FWA00006790) and West Virginia University (FWA00005078), with the Coordinating Center approval at the University of Pittsburgh (FWA00006790). CCDG: COHRA1/Dental SCORE and CCDG: COHRA2/COHRA Smile were supported by US National Institutes of Health (NIH) grants R01-DE014899 and X01-HG009878-01.

**Iowa Fluoride Study**

The Iowa Fluoride Study cohort (IFS; Levy et al. 2013, Wang et al. 2012) began with families from eight postpartum hospitals in Iowa, accounting for approximately 20% of the Iowa births. Participating cohort members underwent frequent questionnaire data collection and several clinical examinations, and questionnaire data collection, beginning at average age 5 years, then also at average ages 9, 13, 17, and 23 years. For purposes of the current study which focuses on adults 18 years or older, data from the average age 23 longitudinal time points were included. The IFS study protocol included longitudinal investigations of fluoride, dietary exposures, and oral health. Standardized caries exams without radiographs of the permanent dentition were conducted at each longitudinal time point, with exams of the permanent dentition at age 23 the focus here. Questionnaires were sent at-least semi-annually to assess fluoride exposures and intakes, select food and beverage intakes, and tooth brushing frequency. All components of the IFS were approved by the Institutional Review Board of the University of Iowa (FWA000003007). CCDG: IFS was supported by NIH grants R01-DE09551, X01-HG008978, R01-DE014899, and P30-DE10126

**CCDG: OFC1/OFC2**

The OFC1 and OFC2 study cohorts come from the University of Pittsburgh Center for Craniofacial and Dental Genetics (CCDG)´s, Orofacial Clefts Study. The POFC study populations were recruited from multiple cleft centers in the United States, including in the states of Colorado, Iowa, Pennsylvania, Texas, and Puerto Rico, and internationally, from Argentina, the Philippines, and Hungary for the purpose of studying orofacial clefts (Leslie et al. 2016). Participants of all ages are included in the cohorts, but for the current study only adults 18 years or older were included. The same data collection protocols were used for every site. Dental caries scores (dft/DFT) were assessed by trained dentists or dental hygienists, with data collected either through in-person dental examinations and/or intraoral photos (at least five photos per subject, maxillary and mandibular occlusal, right and left lateral, anterior biting) to appropriately cover the entire oral cavity, as previously reported (Howe et al. 2017). The DFT/dft indices were calculated as the total number of teeth with decayed, and/or filled/restored surfaces. In addition, questionnaires recording dental history were collected for all participants in the study. All training and calibration for the intra-oral photos and the in-person dental exams was done at the University of Pittsburgh before the start of data collection. Institutional review board (IRB) approval was obtained at each recruitment site by the appropriate IRB process and committee, with a coordinating IRB at the University of Pittsburgh for oversight (FWA00006790). CCDG: OFC1 and CCDG: OFC2 were supported by NIH grants R01-DE016148, X01-HG00784, and X01-HG011437.

**Supplemental Methods**

**Variation in dental caries measurement**

Streamlining dental caries experience analyses on such a large scale, while a unique opportunity, can be daunting. First, variation exists in what has been measured and how in terms of caries experience. In the Hispanic Community Health Study/Study of Latinos (HCHS/SOL), caries experience was recorded at the tooth surface-level by calibrated dental examiners and well-defined criteria previously used in national surveys (Morrison et al. 2016). In the Malmö Diet Cancer (MDC) Study and the Malmö Offspring Study (MOS), dental caries experience was recorded by dentists and data were extracted from their charts. Meanwhile, in the Pittsburgh Center for Craniofacial and Dental Genetics (CCDG) OFC1 and OFC2 cohorts, DMFT indices were derived from intraoral photographs of the permanent dentition, and in Generation Scotland only a binary case status is available, inferred from dental treatment records. Second, variation exists in population demographics (i.e., age, race/ethnicity), as well as disease prevalence and severity.

**Power estimates for detecting genetic associations in GLIDE2 and GLIDE**

Power estimates for genetic main effects were computed using QUANTO (Gauderman, 2002). In GLIDE2, we expect to have high power to detect genetic associations with dental caries (assuming 94.3% caries prevalence, based on the weighted average from cohorts shown in Table 2) for common variants with effect sizes of OR≥1.1, whereas the original GLIDE sample (with available clinical data) was not powered to detect variants with effects of this magnitude. Likewise, in caries severity analyses in GLIDE2 we expect to have high power to detect variants with effect sizes of beta≥0.025 (i.e., per allele difference in units of trait standard deviations), whereas the original GLIDE consortium was insufficiently powered to detect variants of this effect size. Indeed, GLIDE2 has 80% power to detect individual variants explaining 0.008% of variation. For comparison, the original GLIDE had 80% power to detect variants explaining 0.15% of variation – i.e., 18.75 times as large. Altogether, these power estimates show GLIDE2 is poised to have substantially higher power to detect variants with smaller effect sizes and explaining less variation in caries experience, compared with the original GLIDE.

**Supplemental Discussion**

A separate array of harmonization steps and challenges will emerge in the stage of GWAS results meta-analysis, as different cohorts have used different genotyping platforms and are not aligned to a common imputation reference panel. Publicly available data analytic solutions are now available to help overcome such obstacles--for example, the Trans-Omics for Precision Medicine (TOPMed) imputation server (Taliun et al. 2021) contains the most comprehensive and diverse genome imputation references and automates several imputation, quality assessment, and quality control steps. Imputation and harmonization of genomics datasets can be performed in a secure manner and independently by each participating study; for these reasons, it is a logical candidate for harmonizing contributing genomics datasets prior to analysis.

| **Appendix Table 1**. Data sources, availability, and key references (where available) for the 8 cohorts contributing to the caries cluster caries experience analysis in the present paper. | | | | |
| --- | --- | --- | --- | --- |
| **Cohort** | **Dental clinic**  **records** | **Research clinical examinations** | **Data availability** | **References** (PMID) |
| SIMPLER ***S****wedish* ***I****nfrastructure for* ***M****edical* ***P****opulation-Based* ***L****ife-Course and* ***E****nvironmental* ***R****esearch* | X |  | Not publicly available (GDPR protected) | Not available |
| STR *The Swedish Twin Register* | X |  | Not publicly available (GDPR protected) | 31235808, 31905308, 33745184 |
| MDC/MOS *Malmö Diet Study and Malmö Offspring study* | X |  | Not publicly available (GDPR protected) | 31235808 |
| VIKING | X |  | Not publicly available (GDPR protected) | Not available |
| CCDG: COHRA1/ *Dental SCORE*  *(Pittsburgh Center for Craniofacial and Dental Genetics: Center for Oral Health Research in Appalachia Cohort1 plus Dental SCORE)* |  | X | dbGaP*, accession # phs000096.v4.p2 | 18522740 |
| *CCDG: COHRA2/ COHRASmile*  *(Pittsburgh Center for Craniofacial and Dental Genetics: Center for Oral Health Research in Appalachia Cohort 2 plus Dental Score)* |  | X | dbGaP*, accession #phs001591.v1.p1 | 26089906 |
| Periogene North | X |  | Not publicly available (GDPR protected) | Not available |
| IFS  *Iowa Fluoride Study* |  | X | dbGaP*, accession #phs000096.v4.p2 | 12740537, 22508493 |
| *database of genotypes and phenotypes; <https://www.ncbi.nlm.nih.gov/gap/> | | | | |

| **Appendix Table 2.** Tooth surface-level caries experience allocation to 5 caries pattern clusters (Shaffer et al. 2013a), according to the FDI and the Universal tooth identification systems. These cluster definitions were used to score the 8 cohorts contributing caries pattern data in the present study. | | | |
| --- | --- | --- | --- |
| **Trait** | **ID system** | **Contributing surfaces** | **Description** |
| Cluster 1 | FDI | 17-O, 16-OL, 26-OL, 27-O, 37-O, 36-OB, 46-OB, 47-O | molar pits & fissures  (12 surfaces) |
|  | Universal | 2-O, 3-OL, 14-OL, 15-O, 18-O, 19-OB, 30-OB, 31-O |  |
|  |  |  |  |
| Cluster 2 | FDI | 33-MBDL, 32-MDBL, 31-MDBL, 41-MDBL, 42-MDBL, 43-MDBL | lower anterior teeth  (24 surfaces) |
|  | Universal | 22-MBDL, 23-MDBL, 24-MDBL, 25-MDBL, 26-MDBL, 27-MDBL |  |
|  |  |  |  |
| Cluster 3 | FDI | 17-MBDL, 16-BDM, 15-MDO, 14-DO, 24-DO, 25-MDO, 26-BDM, 27-MBDL, 37-MBDL, 36-MDL, 35-DO, 45-DO, 46-MDL, 47-MBDL | molar smooth surfaces, premolar pits and proximal surfaces  (42 surfaces) |
|  | Universal | 2-MBDL, 3-BDM, 4-MDO, 5-DO, 12-DO, 13-MDO, 14-BDM, 15-MBDL, 18-MBDL, 19-MDL, 20-DO, 29-DO, 30-MDL, 31-MBDL |  |
|  |  |  |  |
| Cluster 4 | FDI | 12-MBDL, 11-MBDL, 21-MBDL, 22-MBDL | maxillary incisors  (16 surfaces) |
|  | Universal | 7-MBDL, 8-MBDL, 9-MBDL, 10-MBDL |  |
|  |  |  |  |
| Cluster 5 | FDI | 15-BL, 14-MBL, 13-MBDL, 23-MBDL, 24-MBL, 25-BL,  35-MBL, 34-MBLDO, 44-MBDLO, 45-MBL | maxillary canines and premolar smooth surfaces (34 surfaces) |
|  | Universal | 4-BL, 5-MBL, 6-MBDL, 11-MBDL, 12-MBL, 13-BL,  20-MBL, 21-MBLDO, 28-MBDLO, 29-MBL |  |

**Appendix References**

Gauderman WJ. 2002. Sample size requirements for matched case-control studies of gene-environment interaction. Stat Med. 21(1):35-50.

Howe BJ, Cooper ME, Wehby GL, Resick JM, Nidey NL, Valencia-Ramirez LC, Lopez-Palacio AM, Rivera D, Vieira AR, Weinberg SM, et al. 2017. Dental Decay Phenotype in Nonsyndromic Orofacial Clefting. J Dent Res. 96(10):1106-1114.

Leslie EJ, Carlson JC, Shaffer JR, Feingold E, Wehby G, Laurie CA, Jain D, Laurie CC, Doheny KF, McHenry T, et al. 2016. A multi-ethnic genome-wide association study identifies novel loci for non-syndromic cleft lip with or without cleft palate on 2p24.2, 17q23 and 19q13. Hum Mol Genet. 25(13):2862-2872.

Levy SM, Warren JJ, Broffitt B, Hillis SL, Kanellis MJ. 2003. Fluoride, beverages and dental caries in the primary dentition. Caries Res. 37(3):157-165.

Manjer J, Carlsson S, Elmståhl S, Gullberg B, Janzon L, Lindström M, Mattisson I, Berglund G. 2001. The Malmö Diet and Cancer Study: representativity, cancer incidence and mortality in participants and non-participants. Eur J Cancer Prev. 10:489-499.

Morrison J, Laurie CC, Marazita ML, Sanders AE, Offenbacher S, Salazar CR, Conomos MP, Thornton T, Jain D, Laurie CA, et al. 2016. Genome-wide association study of dental caries in the Hispanic Communities Health Study/Study of Latinos (HCHS/SOL). Hum Mol Genet. 25(4):807-816.

Neiswanger K, McNeil DW, Foxman B, Govil M, Cooper ME, Weyant RJ, Shaffer JR, Crout RJ, Simhan HN, Beach SR, et al. 2015. Oral Health in a Sample of Pregnant Women from Northern Appalachia (2011-2015). Int J Dent. 2015:469376.

Polk DE, Weyant RJ, Crout RJ, McNeil DW, Tarter RE, Thomas JG, Marazita ML. 2008. Study protocol of the Center for Oral Health Research in Appalachia (COHRA) etiology study. BMC Oral Health. 8:18.

Taliun D, Harris DN, Kessler MD, Carlson J, Szpiech ZA, Torres R, Taliun SAG, Corvelo A, Gogarten SM, Kang HM, et al. 2021. Sequencing of 53,831 diverse genomes from the NHLBI TOPMed Program. Nature. 590(7845):290-299.

Wang X, Willing MC, Marazita ML, Wendell S, Warren JJ, Broffitt B, Smith B, Busch T, Lidral AC, Levy SM. 2012. Genetic and environmental factors associated with dental caries in children: the Iowa Fluoride Study. Caries Res. 46(3):177-184.
